# Sonoelectrochemical mineralization of perfluorooctanoic acid using *Ti/PbO*_*2*_ anode assessed by response surface methodology

**DOI:** 10.1186/s40201-015-0232-9

**Published:** 2015-11-14

**Authors:** Gholamreza Bonyadinejad, Mohsen Khosravi, Afshin Ebrahimi, Roya Nateghi, Seyed Mahmood Taghavi-Shahri, Hamed Mohammadi

**Affiliations:** Environment Research Center and Department of Environmental Health Engineering, School of Health, Isfahan University of Medical Sciences (IUMS), Isfahan, 81676-36954 Iran; Student Research Center, School of Health, IUMS, Isfahan, Iran; Nanotechnology Department, University of Isfahan, Isfahan, 81744-73441 Iran; Research Center for Environmental Pollutants, Qom University of Medical Sciences, Qom, 37136-49373 Iran

**Keywords:** Ultrasonics, Lead dioxide, Perfluorooctanoic acid, Central composite design

## Abstract

**Background:**

Perfluorocarboxylic acids (PFCAs) are emerging pollutant and classified as fully fluorinated hydrocarbons containing a carboxylic group. PFCAs show intensively resistance against chemical and biological degradation due to their strong C–F bond. The Sonoelectrochemical mineralization of the synthetic aqueous solution of the perfluorooctanoic acid (PFOA) on Ti/PbO_2_ anode was investigated using the response surface methodology based on a central composite design with three variables: current density, pH, and supporting electrolyte concentration.

**Methods:**

The defluorination ratio of PFOA was determined as an indicator of PFOA mineralization. Fluoride ion concentration was measured with an ion chromatograph unit. The Ti/PbO2 electrode was prepared using the electrochemical deposition method. The ultrasonic frequency was 20 kHz.

**Results:**

The optimum conditions for PFOA mineralization in synthetic solution were electrolyte concentration, pH, and current density of 94 mM, 2, and 83.64 mA/cm^2^, respectively. The results indicated that the most effective factor for PFOA mineralization was current density. Furthermore, the PFOA defluorination efficiency significantly enhanced with increasing current density. Under optimum conditions, the maximum mineralization of PFOA was 95.48 % after 90 min of sonoelectrolysis.

**Conclusions:**

Sonoelectrolysis was found to be a more effective technique for mineralization of an environmentally persistent compound.

## Introduction

The Perfluorocarboxylic acids are classified as fully fluorinated hydrocarbons materials that contain a carboxylic group. Due to their high surface active nature, high thermal and chemical stabilities, PFCAs are extensively using in fire retardants, industrial surfactants and in fluoropolymers manufacturing processes [1]. This extensive utilization of PFCAs results in releasing of these compounds into the environment, which was estimated at 3200–7300 tons for the period of 1950–2004 by direct and indirect emissions [2]. PFCAs show intensive resistance against chemical and biological degradation. Conventional treatment methods are ineffectual for the degradation of PFCAs because it is intrinsically recalcitrant to biological and conventional chemical treatment [3]. The marvelous stability of perfluorinated compounds (PFCs) is ascribed to their strong C–F bond which makes them very persistent to most natural conditions [1, 4]. Perfluorooctanoic acid (PFOA) is one of the PFCAs’ family, which is categorized as a likely potential carcinogen by The US EPA’s Science Advisory Board in 2006. Recently, PFOA and its precursors have been universally detected in wildlife, water and human body [5]. For instance, the river Elbe in Germany was polluted by PFOA due to effluents discharged from wastewater treatment plant [6]. Since, PFOA has toxicity to organisms and humans and persistence in the environment, a lot attention has been paid to treatment of PFOA from water which uses effective methods under moderate conditions. Lately, some chemical technologies for PFOA degradation have been reported, such as photocatalytic oxidation, direct photolysis, photochemical reduction, photochemical oxidation, thermally-induced reduction and sonochemical pyrolysis. Yet, most of the techniques could not effectively decompose PFOA [7]. The goal of destructive methods is cleaving the C–F bonds to form F- ions [2]. The electrochemical oxidation is a technology that has presented its capacity to degrade refractory organic pollutants such as emerging contaminants contained in the secondary effluents of wastewater treatment plants and also removal of various pollutants from aqueous environments [8, 9]. Electrochemical techniques generally carried out oxidatively, have the advantage of contaminant elimination without the addition of chemicals. Although electrochemical methods are easy to use and have high removal efficiency, energy consumption is the main disadvantage of these methods. Nevertheless, this disadvantage has been suppressed by the development of new anode materials [10, 11]. PbO_2_ is a low-cost electrode material that can be quickly and easily prepared, and is being used by many researchers for electro-oxidation of refractory pollutants in aquatic solutions [12–15]. During the process of oxidation of polluted waters, OH^•^ specimen is generated on the PbO_2_ electrode surface, and a mechanism for the electrochemical processes occurring in the gel-crystal structure of the PbO_2_ layer of the electrode has been proposed [16]. The Pb*O(OH)_2_ active centers placed in the hydrated PbO_2_ layer on the surface of the crystalline PbO_2_ anode provide electrons to the crystal district, becoming positively charged (Pb*O(OH)^+^ (OH)^•^). This electric charge is neutralized according to the following reaction:

1 $$ \mathrm{P}\mathrm{b}\ast \mathrm{O}{\left(\mathrm{O}\mathrm{H}\right)}^{+}{\left(\mathrm{O}\mathrm{H}\right)}^{\bullet }+{\mathrm{H}}_2\mathrm{O}\;\to\;\mathrm{P}\mathrm{b}\mathrm{O}{\left(\mathrm{O}\mathrm{H}\right)}_2\dots {\left(\mathrm{O}\mathrm{H}\right)}^{\bullet }+{\mathrm{H}}^{+} $$

in which hydroxyl radicals are produced in the active centers. The OH• can depart from the active centers and react with the pollutant in the aqueous solution. Thus, the PbO_2_ anode is expected to perform quite well in organic pollutant mineralization. However, the main problem of PbO_2_ anode is the release of poisonous ions, Pb^2+^ [17]. On the other hand, sonochemical treatment applies pyrolytic cleavages for organic pollutants degradation and is an emerging and impressive method which can be used efficiently to eliminate PFOA particularly [18]. Ultrasonic (US) treatment acts as cavitation, which not only generates plasma in water, and degrading molecules by pyrolysis, but also produces free radicals and other reactive types that can improve the amount of collisions between free radicals and contaminants. Recently, using the combination of ultrasonic method and other techniques for the treatment of organic wastewater has been largely studied. (e.g. phenol and pharmaceutical compounds) [19–21]. Optimization of the operating conditions of an experimental system and recognition of the way in which the experimental parameters affect the final output of the system are realized by using modeling techniques [22]. In addition, it is possible to determine the relationships and interactions between the variables through these techniques. In this regard, statistical methodologies, such as the response surface methodology (RSM), are suitable for studying and modeling a particular system [23]. The aim of the present study was to investigate the electrochemical mineralization of PFOA as an emerging contaminant using Ti/PbO_2_ anode coupled with ultrasonic irradiation (sonoelectrochemical) and determine the optimum conditions by RSM. The effects of current density (CD), pH of the solution, and supporting electrolyte (EL) concentration were evaluated in terms of PFOA defluorination.

## Materials and methods

### Chemicals

Analytical-grade PFOA was purchased from Sigma Aldrich co., and used without further purification. Pb(NO_3_)_2_ (Sigma Aldrich co.), Triton X-100 (Merck co.), and CuSO_4_.5H_2_O (Merck co.) were used for electrode preparation. Other chemicals were purchased from Merck co. The initial pH of the solutions was adjusted using sodium hydroxide and sulfuric acid. Sodium sulfate was used as the supporting electrolyte. All the solutions were prepared using de-ionized water.

### Preparation of Ti/PbO_2_ electrode

The Ti substrate with 2 mm thickness was cut into a strip (4.8 cm × 4 cm, 99.7 % Aldrich) and pre-treated according to the following procedure: the substrate was polished on 320-grit paper strips [24] to eliminate the superficial layer of TiO_2_ (an electric semiconductor) and increase surface roughness (for efficient adherence of PbO_2_). Then, the substrate was degreased in an ultrasonic bath of acetone for 10 min and then in distilled water for 10 min. Afterwards, the substrate was etched for 1 h in a boiling solution of oxalic acid (10 %) and rinsed with ultrapure water [25]. Finally, the cleaned Ti substrate was transferred to an electrochemical deposition cell, which contained 12 % (w/v) Pb(NO_3_)_2_ solution comprising 5 % (w/v) CuSO_4_.5H_2_O and 3 % (w/v) surfactant (Triton X-100). The role of the surfactant was to minimize the surface tension of the solution for better wetting of the substrate and also to increase the adhesion of PbO_2_ to the Ti substrate. The electrodeposition of PbO_2_ was performed at a constant anodic current of 20 mA/cm^2^ for 60 min at 80 °C with continuous stirring [26]. The X-ray diffraction (XRD) tests were performed using a Bruker, D8 Advance, Germany. The samples were scanned under Co Kα radiation (wavelength: 1.7890 Å) at 40 kV and 40 mA. Scanning electron microscope (SEM; Philips XI30, Netherlands) was employed to observe the surface morphology of the electrodes, which presented a typical pyramid shape similar to that reported in the literature [27].

### Sonoelectrochemical mineralization of PFOA

**Fig. 1:**
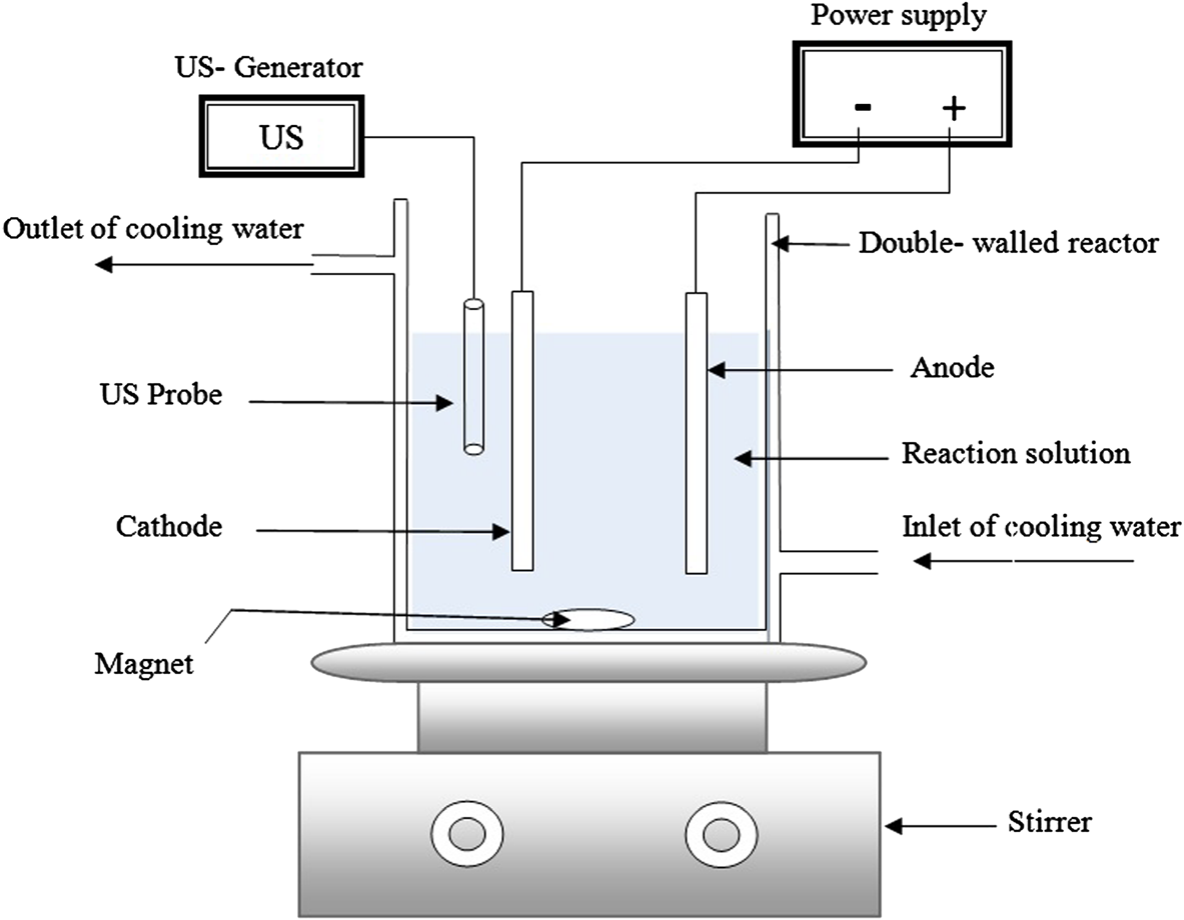
Schematic of the experimental setup

Synthetic wastewater was prepared by dissolving PFOA in distilled water at a concentration of 50 mg/L. The sonoelectrochemical mineralization of PFOA was performed in a temperature-controlled water batch reactor (0.45 L) equipped with an ultrasonic probe (Bandelin SONOPULS,UW 3200, TT 13, Germany), a 41.12 cm^2^ Ti/PbO_2_ as the anode and a 80.32 cm^2^ stainless steel plate as the cathode in conjunction with an adjustable power supply unit (HANI, Iran). The gap between the anode and cathode was 1 cm. The temperature of the reaction solution was kept constant at 25 ± 1 °C. The duration of all the electrolysis experiments was 90 min. The reactor was placed on a magnetic stirrer to mix its content during the experiment in order to maximize mass transport (Fig. 1).

### Analytical procedure

Defluorination ratio of PFOA was determined based on the release of fluoride into the solutions. The defluorination ratio, is the indicator of PFOA mineralization [28]. Fluoride ions concentration were measured with an Ion chromatograph system (IC762, Metrohm, USA) equipped with an automatic sample injector, a degasser, a pump, a guard column (Metrosep RP guard, Metrohm), a separation column (Metrosep Anion Dual 2, Metrohm), and a conductivity detector with a suppressor device. The mobile phase was an aqueous solution containing NaHCO_3_ (2 mM) and Na_2_CO_3_ (1.3 mM). The flow rate was 0.8 mL/min. The defluorination ratio (R) was calculated as Eq. 2:

2 $$ \mathrm{R}=\frac{{\mathrm{C}}_{\mathrm{F}}\hbox{-} }{{\mathrm{C}}_0\times 15}\times 100 $$

where C_F_- is the concentration of fluoride in mM, C_0_ is the initial concentration of PFOA in mM, and the value of 15 represents the number of fluorine atoms contained in one PFOA molecule.

### Experimental design

The central composite design (CCD) technique coupled with RSM has been opted for modeling and design of experimental tests [29]. The CD, EL, and pH parameters were selected as input variables. The rotatable experimental plan was performed with the three variables at five levels (−1.68, −1, 0, 1, 1.68). Table 1 shows the values and levels of the variables. Five replications were done at the center point of the design to evaluate the pure error and consequently the lack of fit. Statistica ver. 10, and R ver. 3.1.2 software were used to design and analyze the experiments. Table 2 shows the CCD matrix of the mineralization experiments.

**Table 1:** The range and codification of the independent variables (X_i_) used in the experimental design

Variables	Actual values of the coded values				
	−1.68	−1	0	1	1.68
pH (X_1_)	1.95	4	7	10	12.05
EL (mM) (*X*_2_)	32.96	50	75	100	117.04
CD (mA/cm^2^) (X_3_)	16.36	30	50	70	83.64

**Table 2:** CCD matrix of sonoelectrochemical mineralization of PFOA

Exp. No.	pH	EL	CD	Defluorination ratio (%)	
				Observed	Predicted
1	4.00	50.00	30.00	54.16	41.90
2	4.00	50.00	70.00	87.45	81.34
3	4.00	100.00	30.00	57.47	44.16
4	4.00	100.00	70.00	89.49	83.61
5	10.00	50.00	30.00	51.89	38.57
6	10.00	50.00	70.00	85.55	78.03
7	10.00	100.00	30.00	51.46	40.84
8	10.00	100.00	70.00	87.46	80.29
9	1.95	75.00	50.00	75.71	67.15
10	12.05	75.00	50.00	69.6	61.57
11	7.00	32.96	50.00	69.12	61.36
12	7.00	117.04	50.00	74.26	65.18
13	7.00	75.00	16.36	37.73	23.20
14	7.00	75.00	83.64	91.86	89.55
15	7.00	75.00	50.00	74.32	65.36
16	7.00	75.00	50.00	75.51	65.36
17	7.00	75.00	50.00	74.66	65.36
18	7.00	75.00	50.00	73.82	65.36
19	7.00	75.00	50.00	74.32	65.36

The relationship between response Y and the three independent variables X_1_, *X*_2_, and X_3_ could be approximated by quadratic polynomial Eq. 3 as follows:

3 $$ \mathrm{Y}={\mathrm{b}}_0+{\mathrm{b}}_1{\mathrm{X}}_1+{\mathrm{b}}_2{\mathrm{X}}_2+{\mathrm{b}}_3{\mathrm{X}}_3+{\mathrm{b}}_{11}{{\mathrm{X}}_1}^2+{\mathrm{b}}_{22}{{\mathrm{X}}_2}^2+{\mathrm{b}}_{33}{{\mathrm{X}}_3}^2+{\mathrm{b}}_{12}{\mathrm{X}}_1{\mathrm{X}}_2+{\mathrm{b}}_{13}{\mathrm{X}}_1{\mathrm{X}}_3+{\mathrm{b}}_{23}{\mathrm{X}}_2{\mathrm{X}}_3 $$

where Y is the predicted response; b_0_ is a constant; b_1_, b_2_, and b_3_ are the linear coefficients, b_12_, b_13_, and b_23_ are the cross-product coefficients; and b_11_, b_22_, and b_33_ are the quadratic coefficients. In the present study, backward variable selection was used for multiple regression modeling [30]. The assumption of final regression model was verified using the Anderson–Darling test for normality of residuals [31], Breusch–Pagan test for constant variance of residuals [32], and Durbin–Watson test for independence of residuals [33, 34]. Lack of fit test was performed to assess the fit of the final model. Validation of the final model was established using predicted R-squares (R^2^), which estimates the prediction power of the model with new observations based on the leave-one-out technique [35]. The optimum values of the final model were calculated using numerical methods. In this regard, the experimental range predictors were divided into a grid and then the final model was calculated for all possible combinations of predictors in the grid.

## Result and discussion

### Characterization of the Ti/PbO_2_ electrode

**Fig. 2:**
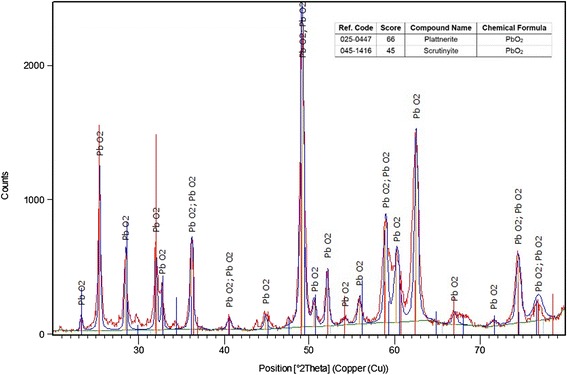
XRD pattern of the prepared PbO_2_ electrode

**Fig. 3:**
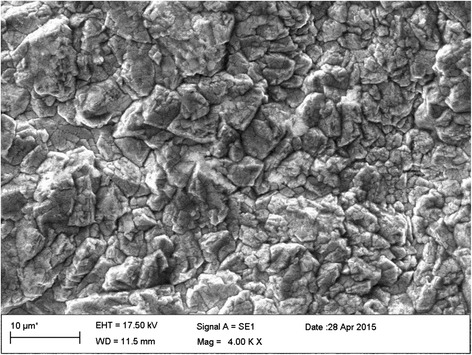
SEM micrograph of the surface of the Ti/PbO_2_ electrode

XRD pattern of the Ti/PbO_2_ electrode has been represented in Fig. 2, it can be observed that PbO_2_ was deposited in the form of two known polymorphs, namely, orthorhombic α-PbO_2_ and tetragonal β-PbO_2,_ which occur naturally as scrutinyite and plattnerite, respectively. Figure 3 shows the SEM image of the surface microstructure of the Ti/PbO_2_ electrode. It can be observed that the PbO_2_ layer is crack free and composed of packed faceted micro crystallites. Such morphology verifies that only PbO_2_ is involved in the electrochemical mineralization of the PFOA and protects the surface of the Ti substrate. Furthermore, energy-dispersive X-ray spectroscopy (EDS) analysis (data not shown) confirmed the presence of lead and oxygen atoms on the surface of the Ti/PbO_2_ electrode.

### CCD analysis and modeling

The interaction effect of input variables, which were statistically designed by using CCD method were studied through different combination of experimental parameters. The CCD design matrix, the predicted results and observed defluorination ratio values for the sonoelectrochemical mineralization of PFOA are shown in Table 2. Equation 4 represents the first model that was developed with all linear, quadratic, and two-way interaction of predictors:

4 $$ \mathrm{Y}=6.04096\;\hbox{-}\;0.045\times \mathrm{p}\mathrm{H}+0.267\times \mathrm{EL}+1.6625\times \mathrm{C}\mathrm{D}\;\hbox{-}\;0.00565\times \mathrm{p}\mathrm{H}\times \mathrm{EL}+0.01108\times \mathrm{p}\mathrm{H}\times \mathrm{C}\mathrm{D}+0.000106\times \mathrm{EL}\times \mathrm{C}\mathrm{D}\hbox{-} 0.04639\times {\mathrm{pH}}^2\;\hbox{-}\;0.001247\times {\mathrm{EL}}^2\;\hbox{-}\;0.008789\times {\mathrm{CD}}^2 $$

The predicted R^2^ of this initial model was 98.0 %. Backward elimination method was used to achieve a parsimonious model with significant predictors. In the first step, linear form of pH was removed from the initial model (*p* = 0.88). In three following steps, interactions of EL*CD (*p* = 0.73), pH*EL (*p* = 0.17), and pH*CD (*p* = 0.33) were removed from the model. Finally, linear and quadratic form of EL and CD, with quadratic form of pH, EL, and CD remain significant in the prediction model. The final model was as Eq. 5:

5 $$ \mathrm{Y}=5.069+0.22311\times \mathrm{EL}+1.62182\times \mathrm{C}\mathrm{D}\;\hbox{-}\;0.039453\times {\mathrm{pH}}^2\;\hbox{-}\;0.0011852\times {\mathrm{EL}}^2\;\hbox{-}\;0.0079442\times {\mathrm{CD}}^2 $$

**Fig. 4:**
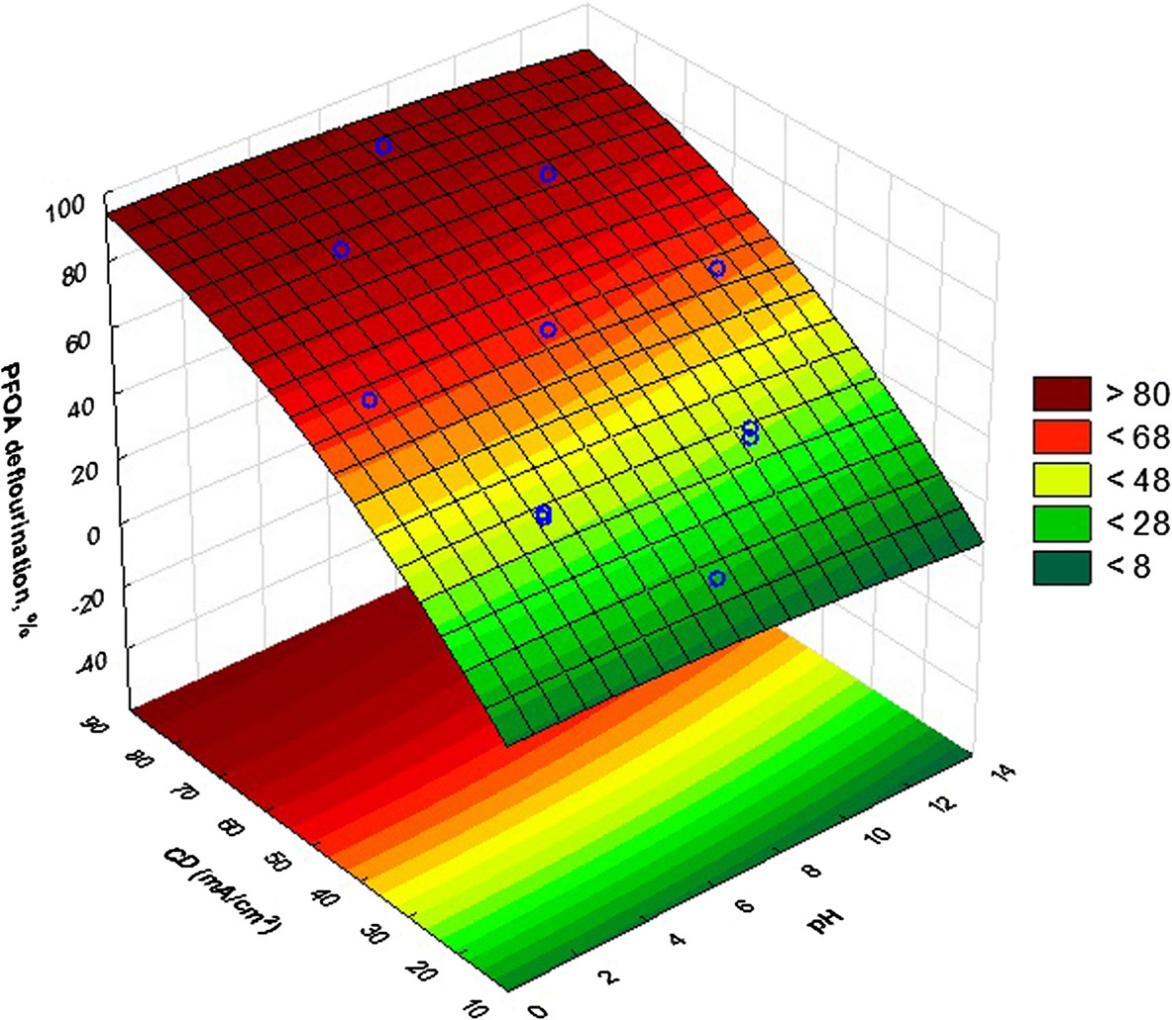
3D surface plot for the PFOA deflourination as a function of CD and pH

A partial F test was performed for comparison between first model and the final model. The value of F was 0.86 with 4 and 9° of freedom, and a P-value of 0.52. Hence, the difference of these two models was not significant, although the final regression model (Eq. 5) had four predictors less than the initial model (Eq. 4). Table 3, represent the final regression model for PFOA mineralization. The P-value of Anderson-Darling, Breusch-Pagan, and Durbin-Watson tests were 0.34, 0.82 and 0.75, respectively, which confirmed the assumptions of regression model. Lack of fit test also was non-significant with P-value of 0.09 which means the final model fitted experimental data satisfactorily. (F = 4.08, degree of freedom for lack of fit = 9, degree of freedom for pure error = 4) (Fig. 4).

**Table 3:** Final regression model for PFOA mineralization

Variable	Coefficients	Std. Error	T statistic	P-value
(Constant)	5.07	3.36	1.51	0.155
EL	2.23 × 10^−1^	7.23 × 10^−2^	3.09	0.009
CD	1.62	7.57 × 10^−2^	21.42	<0.001
pH^2^	−3.95 × 10^−2^	6.98 × 10^−3^	−5.65	<0.001
EL^2^	−1.18 × 10^−3^	4.75 × 10^−4^	−2.49	0.027
CD^2^	−7.94 × 10^−3^	7.42 × 10^−4^	−10.71	<0.001

R-squared = 99.6 %, Adjusted R-squared = 99.4 %, Predicted R-squared = 98.8 %

**Fig. 5:**
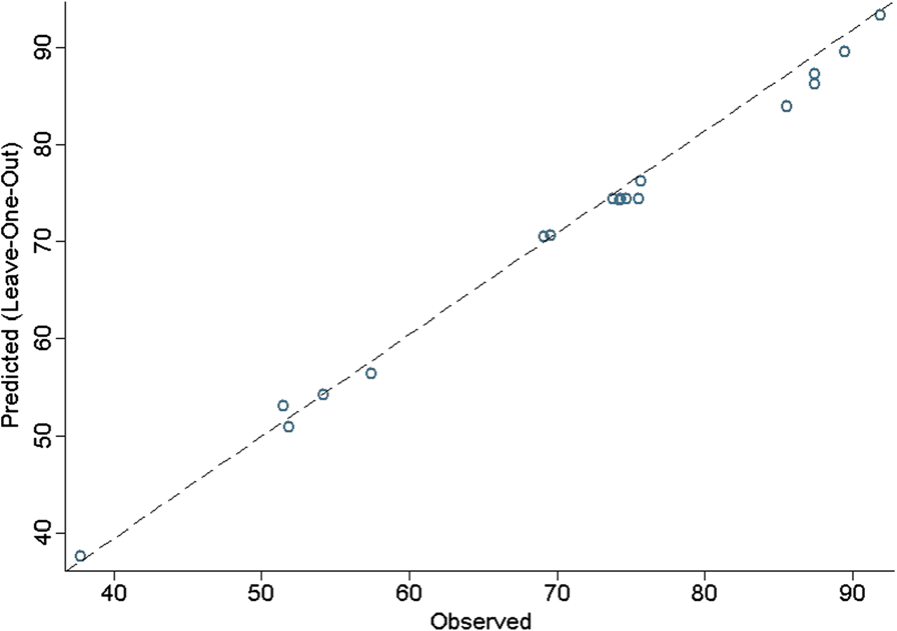
Leave-one-out prediction of the final model vs. observed for PFOA mineralization

The final model was validated using leave-one-out technique. Predicted R-squared was 98.8 % which confirmed external validity of the final model. Leave-one-out prediction of the final model vs. observed values of response represented in Fig. 5.

**Fig. 6:**
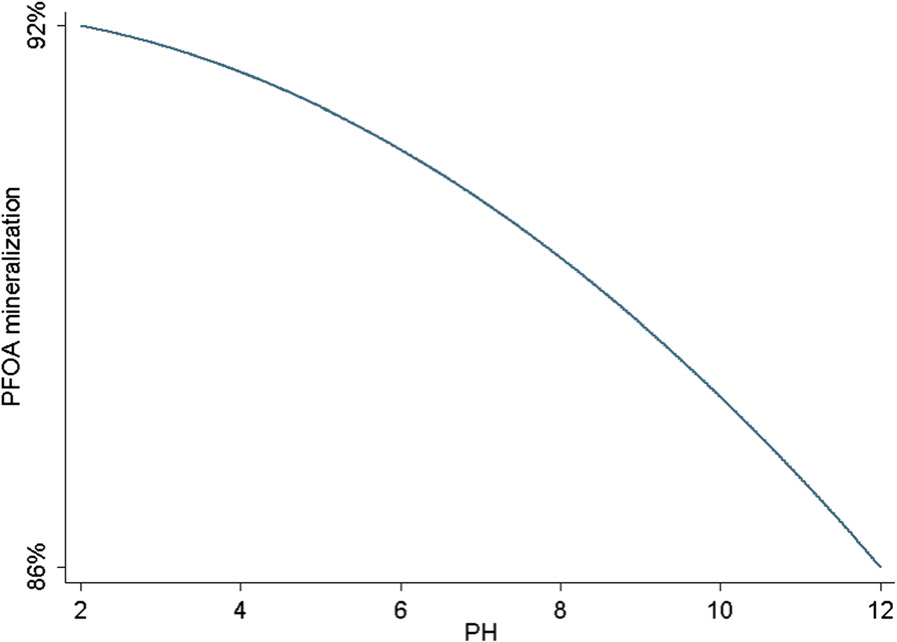
Predicted PFOA mineralization using the final model vs. pH, when EL = 94 mM and CD = 83.64 mA/cm^2^

**Fig. 7:**
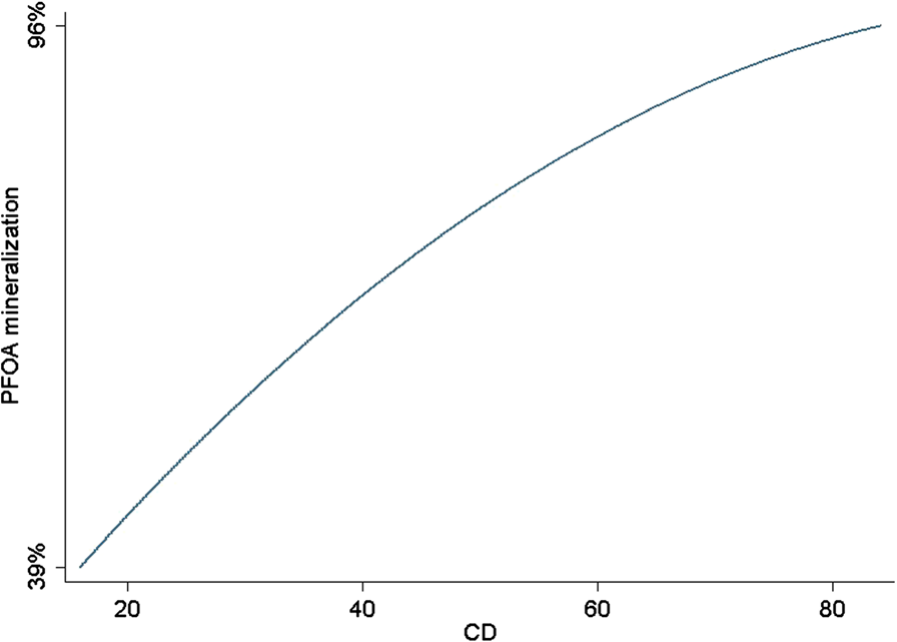
Predicted PFOA mineralization using the final model vs. CD, when pH = 2 and EL = 94 mM

**Fig. 8:**
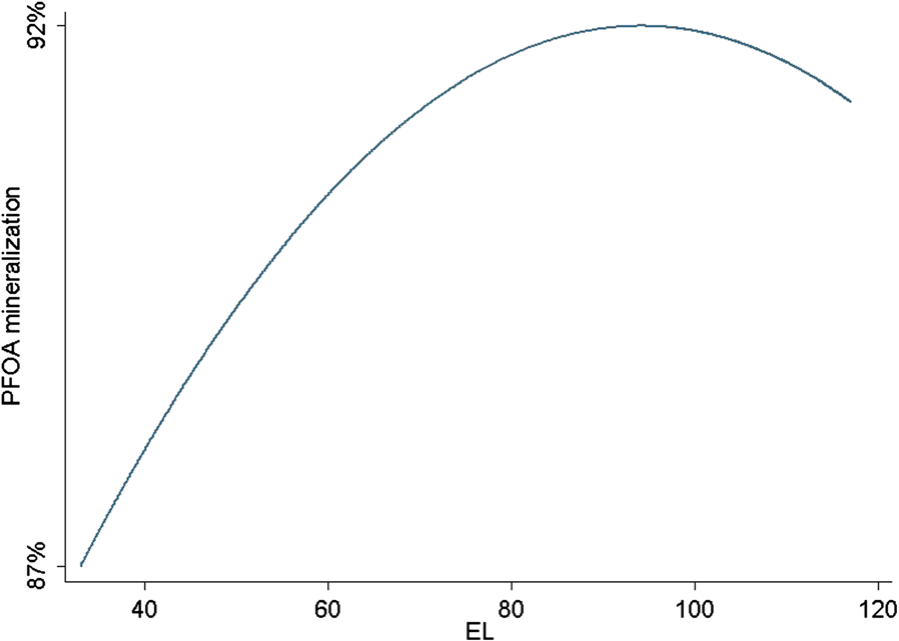
Predicted PFOA mineralization using the final model vs. EL, when pH = 2 and CD = 83.64 mA/cm^2^

The final model was evaluated through the range of experimental predictors with a numerical method. The grid combination of predictors was ranged between 2 to 12 with 0.1 increments for pH, from 33 to 117 with 1 increments for EL and from 16 to 85 with 1 increments for CD. Model prediction was calculated for 579,600 different combination of predictors and optimum values of input parameters were pH = 2, EL = 94, CD = 83.64 which resulted in 95.48 % PFOA mineralization. Since, there are no interaction effects in the final model, effects of pH, EL, and CD on PFOA mineralization can be investigated independently. Figures 6, 7, and 8 show effects of pH, CD, and EL on PFOA mineralization, respectively, when other factors were at optimum values.

### Synergistic effect

In order to evaluate the simultaneous effect of ultrasonic and electrochemical processes on PFOA mineralization, three different pretests for PFOA mineralization were done as follow: sonolysis, electrochemical and sonoelectrochemical. In all three experiments, reaction time, PFOA concentration and initial pH value were 90 min, 50 mg/L, and 7, respectively. The mineralization results were 8 %, 21 % and 73.9 % for sonolysis (frequency = 20 khz), electrochemical (CD = 50, mA/cm^2^, and EL = 75, mM) and sonoelectrochemical (frequency = 20 khz, CD = 50 mA/cm^2^, and EL = 75, mM) processes, respectively. This indicates that combination of sonolysis and electrochemical, have a remarkable synergistic effect on PFOA mineralization. Some of the basic concepts are mentioned here to clarify the sonoeletochemical, results in a higher mineralization efficiency than sonolysis and electrochemical. In the sonolysis process, the propagation of ultrasound waves through the bulk of liquid cause cavitational bubbles, which can oxidize organic substances either directly by formation of •OH by the sonolysis of water, or as a result of thermolytic reactions taking place inside [36]. The ultrasonic technique is powerful, however with respect to total input energy, there is no economical justification for using this method without the help of other techniques [37].

The synergism observed between sonolysis and electrochemical oxidation can be associated with following reasons:

1. Formation of sulfate radical by ultrasonic irradiation in the presence of sulfate ions when sodium sulfate used as supporting electrolyte [38].
2. Ultrasonic waves facilitated the mass-transfer on the electrode surface, resulted in increasing diffusion of the produced hydroxyl radicals,which increased the OH radical concentration in the solution [36, 39, 40].
3. Cleaning of the electrode surface by cavitational bubbles. The mechanical effects of cavitation lead to cleaning of the electrode surface and inhibit any passive layer formation. This effect guarantee the normal electrochemical operation process with a stable electric current in the period of the treatment time [36, 40]

### Effect of initial pH

Figure 4 shows the effect of initial pH of the solutions on PFOA mineralization which has been adjusted to the following values: 1.95, 4, 7, 10, and 12.05. There are many disagreements about the mechanism of influence of pH in the literature which is due to diversity of the organic structures and electrode materials, however the initial pH is one of the main factors in the oxidation process [27].

As shown in Fig. 4 and by Eq. 5, the PFOA mineralization efficiency was increased with decrease of pH. From Eq. (3), the highest and the lowest level of PFOA mineralization efficiency can be reached at an acidic (pH = 2) and alkaline (pH = 12) conditions, respectively. An increase in the PFOA mineralization with the decreasing pH from 12 to 2 can be explained as follows; first, in the alkaline conditions, sulfate radicals might react with OH resulting O^−^ which has a lower oxidation potential lead to PFOA mineralization rate decrease [38]. Second, the enhancement of the PFOA mineralization efficiency at pH values lower than neutral is due to the increase in oxygen over-potential that abate the anodic oxygen evolution reaction and favors the production of more potent oxidizers such as OH radicals that are appropriate for the oxidation of organic compounds [41–43]. Third, many micro bubbles formed at alkaline pH in the aqueous solution in contrast with the amounts of bubbles formed at acidic pH. This situation leads to adherence of bubbles to the sonicator's probe and therefore restrict ultrasound energy distribution through the bulk of solution [37].

However, in the strong acidic solutions, the life of anode decreases [44]. As a result, in the present study, a pH of 2 was found as the optimum pH value for maximum PFOA mineralization. Other researchers also reported similar results [45]. Equation 5 and Fig. 6 show that the difference between the minimum and maximum PFOA mineralization efficiency related to pH is 6 %, which indicates sonoelectrochemical mineralization of PFOA using PbO_2_ anode is not very sensitive to the initial pH and PFOA could be mineralized under a broad range of pH. Therefore, within the scope of the present study, it can be suggested that pre-adjustment of pH with the addition of chemicals is not necessary for sonoelectrochemical mineralization of the studied compound in the full scale treatment, unless a little increase in the mineralization efficiency is logical.

### Effect of CD

In the present study, the effect of CD was investigated at five levels (16.36, 30, 50, 70, and 83.64 mA/cm^2^) in combination with pH and EL (Fig. 4). As shown in this figure, which is the output of the CCD, the PFOA mineralization efficiency significantly increased with the increasing CD. Respect to Eq. (5), the maximum PFOA mineralization efficiency was obtained at the CD of 83.64 mA/cm^2^ in the range of investigation. Equation (5) and Fig. 7 show that the difference between the minimum and maximum PFOA mineralization efficiency related to CD is 57 %, indicating that, the most important variable for the enhancement of PFOA mineralization was CD. The main reason for the increase in efficiency with increasing current density can be attributed to increasing the number of OH specimen produced which is in well agreement with other studies [42, 44, 46]. As a result, in the present study, a CD of 83.64 mA/cm^2^ was chosen as the optimum CD value for maximum PFOA mineralization.

### Effect of EL

The effect of EL was investigated at five levels (32.96, 50, 75, 100, and 117.04 mM) in combination with pH and CD. It can be concluded from Fig. 8 that despite of increasing PFOA mineralization with increasing the concentration of the EL, its impact is not significant. Many researchers which have used electrochemical process for wastewater treatment, believe that the degradation rate of pollutants is not affected by electrolyte concentration [46–48], however this negligible raise in the efficiency with increasing the concentration of the electrolyte can be explained by increasing the concentration of sulphate radicals, which are generated through the irradiation of sulfate ions by ultrasonic that was mentioned before. Change the electrolyte concentration in the range of tests, leading up to a 5 % change in the PFOA mineralization efficiency.

## Conclusions

In the present study, sonoelectrochemical mineralization of the PFOA was investigated and modeled by employing CCD coupled with RSM for the prediction and optimization of the PFOA mineralization in synthetic wastewater using Ti/PbO_2_ as the anode and stainless steel as the cathode. The use of RSM based on CCD allowed determination of the behavior of the sonoelectrolysis on mineralization, without requiring large number of experiments, and provided sufficient information. Moreover, the CCD facilitates the process of selecting optimum conditions for defluorination. The final model was validated using the leave-one-out technique, and the predicted R^2^ was 98.8, which confirmed the external validity of the model. In addition, lack of fit test was nonsignificant with a P-value of 0.09, which confirmed the fit of the final model. The results of the present study demonstrated that sonolysis and electrochemical (using Ti/PbO_2_ anode) processes are not able to mineralize PFOA significantly and combination of them as sonoelectrochemical process is a suitable and an environment-friendly method for the mineralization of refractory PFOA in aqueous solution.

## Abbreviations

PFOA: Perfluorooctanoic Acid
PFCA: Perfluorocarboxylic acid
AOP: Advanced Oxidation Process
EAOP: Electrochemical Advanced Oxidation Process
EO: Electrochemical Oxidation
CD: Current Density
EL: Supporting Electrolyte
RSM: Response Surface Methodology
CCD: Central Composite Design
SEM: Scanning Electron Microscope
XRD: X-Ray Diffraction
